# *C9ORF72* Repeat Expansion in Australian and Spanish Frontotemporal Dementia Patients

**DOI:** 10.1371/journal.pone.0056899

**Published:** 2013-02-20

**Authors:** Carol Dobson-Stone, Marianne Hallupp, Clement T. Loy, Elizabeth M. Thompson, Eric Haan, Carolyn M. Sue, Peter K. Panegyres, Cristina Razquin, Manuel Seijo-Martínez, Ramon Rene, Jordi Gascon, Jaume Campdelacreu, Birgit Schmoll, Alexander E. Volk, William S. Brooks, Peter R. Schofield, Pau Pastor, John B. J. Kwok

**Affiliations:** 1 Neuroscience Research Australia, Sydney, Australia; 2 School of Medical Sciences, University of New South Wales, Sydney, Australia; 3 Huntington Disease Service, Westmead Hospital, Sydney, Australia; 4 Sydney School of Public Health, University of Sydney, Sydney, Australia; 5 South Australia Clinical Genetics Service, Women's and Children's Hospital, Adelaide, Australia; 6 Department of Paediatrics, University of Adelaide, Adelaide, Australia; 7 Department of Neurogenetics, Kolling Institute of Medical Research, Royal North Shore Hospital and University of Sydney, Sydney, Australia; 8 Neurodegenerative Disorders Research Pty Ltd, Subiaco, Australia; 9 Neurogenetics Laboratory, Division of Neurosciences, Center for Applied Medical Research, University of Navarra, Pamplona, Spain; 10 Department of Neurology, Hospital do Salnés, Pontevedra, Spain; 11 Department of Neurology, Hospital de Bellvitge, Barcelona, Spain; 12 Institute of Human Genetics, University Hospital Ulm, Ulm, Germany; 13 Department of Neurology, Clínica Universidad de Navarra, University of Navarra School of Medicine, Pamplona, Spain; 14 CIBERNED, Centro de Investigación Biomédica en Red de Enfermedades Neurodegenerativas, Instituto de Salud Carlos III, Madrid, Spain; Centre Hospitalier Universitaire Vaudois (CHUV), Switzerland

## Abstract

A hexanucleotide repeat expansion in *C9ORF72* has been established as a common cause of frontotemporal dementia (FTD). However, the minimum repeat number necessary for disease pathogenesis is not known. The aims of our study were to determine the frequency of the *C9ORF72* repeat expansion in two FTD patient collections (one Australian and one Spanish, combined n = 190), to examine *C9ORF72* expansion allele length in a subset of FTD patients, and to examine *C9ORF72* allele length in ‘non-expansion’ patients (those with <30 repeats). The *C9ORF72* repeat expansion was detected in 5–17% of patients (21–41% of familial FTD patients). For one family, the expansion was present in the proband but absent in the mother, who was diagnosed with dementia at age 68. No association was found between *C9ORF72* non-expanded allele length and age of onset and in the Spanish sample mean allele length was shorter in cases than in controls. Southern blotting analysis revealed that one of the nine ‘expansion-positive’ patients examined, who had neuropathologically confirmed frontotemporal lobar degeneration with TDP-43 pathology, harboured an ‘intermediate’ allele with a mean size of only ∼65 repeats. Our study indicates that the *C9ORF72* repeat expansion accounts for a significant proportion of Australian and Spanish FTD cases. However, *C9ORF72* allele length does not influence the age at onset of ‘non-expansion’ FTD patients in the series examined. Expansion of the *C9ORF72* allele to as little as ∼65 repeats may be sufficient to cause disease.

## Introduction

Frontotemporal dementia (FTD) is a clinically diagnosed syndrome including a wide spectrum of features, usually leading to a progressive dementia and often accompanied by motor features including parkinsonian syndromes or amyotrophic lateral sclerosis (ALS). FTD can present clinically with personality and behavioural changes (termed behavioural variant FTD, bvFTD) or language deficits (including syndromes termed semantic dementia (SMD) and progressive non-fluent aphasia (PNFA)) [1]. FTD can also present with memory deficits similar to those observed in Alzheimer's disease (AD), which can complicate diagnosis. Corticobasal syndrome (CBS) and progressive supranuclear palsy syndrome (PSPS) are related clinical diagnoses that show substantial pathological overlap with FTD [2].

The underlying pathology of FTD (termed frontotemporal lobar degeneration, FTLD) includes atrophy often most prominent in the frontal and temporal regions, together with the presence of degenerating neurons with intracellular inclusions that are immunopositive for either microtubule-associated protein tau (classed as FTLD-tau) or ubiquitin (FTLD-U). The ubiquitin-positive inclusions are also positive for either TAR DNA binding protein 43 (FTLD-TDP) or fused in sarcoma protein (FTLD-FUS) [2].

Mutations in the genes encoding tau (*MAPT*) and progranulin (*GRN*) have both been shown to lead to FTD, with *CHMP2B* and *VCP* mutations leading to FTD in rarer cases [3]. Recently, a hexanucleotide repeat expansion in *chromosome 9 open reading frame 72* (*C9ORF72*), a gene of unknown function on chromosome 9p21, was reported as a major cause of disease in Finnish and North American FTD and ALS cohorts [4], [5], and has been observed in FTD and ALS cohorts worldwide [6]. An increased prevalence of psychosis has been observed [7], [8] and parkinsonism has been reported as a common phenotype [9], [10] in *C9ORF72*-positive FTD patients. Single nucleotide polymorphism (SNP) analysis has indicated that most expansion carriers share a common haplotype covering the *C9ORF72* gene and surrounding region, which is tagged by the A allele of SNP rs3849942, and indicates that these individuals are descended from a common ancestor [6], [11]–[13].

Two previous studies have indicated that there is no increased risk of ALS [13] or FTD [8] associated with *C9ORF72* allele lengths below that currently defined as an ‘expansion’ (≥30 repeats). However, it is not yet established whether this is true for all populations. In addition, current polymerase chain reaction (PCR)-based assays cannot distinguish between those subjects with ∼50 repeats and those with larger numbers of repeats: thus it is not yet known what the minimum number of repeats is to cause disease. We therefore examined the frequency of this repeat expansion in a collection of Australian and Spanish FTD patients and compared allele length in non-expansion FTD patients and healthy controls in different populations. We also analysed genomic DNA by Southern blotting to determine the approximate number of repeats in selected expansion carriers. We determined that *C9ORF72* expansion was a frequent cause of disease in our patients, and that repeat numbers as little as ∼65 repeats may be sufficient to cause disease. However, repeats <30 units were not associated with risk of disease or age of onset in non-expansion FTD patients.

## Results

We examined the *C9ORF72* gene in a collection of Australian and Spanish FTD patients (Table 1). We identified *C9ORF72* repeat expansions in 23 of the 190 unrelated patients screened in the two FTD groups (12.1%). The expansion was found in 19.8% (18/91) of the Australian patients and 5.1% (5/99) of the Spanish patients. In the subgroup of patients with a positive family history of early-onset (<65 y) dementia or ALS, the frequency rose to 40.5% (15/37) and 21.4% (3/14), respectively.

**Table 1:** Demographic information of FTD patient groups.

Group	No. cases (M/F)	Age at onset (y) (mean±SD)	Clinical diagnosis	Family history (%)
Australian	91 (53/38)	58.7±11.8	8 bvFTD, 6 CBS, 9 FTD-ALS, 9 FTDP, 50 FTDuns, 5 PNFA, 2 SMD, 2 atypical AD	40.7
Spanish	99 (51/47)*	67.1±10.2	69 bvFTD, 6 FTDm, 4 FTD-ALS, 5 FTDP, 10 PNFA, 5 SMD	14.1

bvFTD = behavioural variant FTD; CBS = corticobasal syndrome; D = death; F = female; FTD-ALS = FTD with amyotrophic lateral sclerosis; FTDP = FTD with parkinsonism; FTDm = FTD with mixed phenotype; FTDuns = FTD otherwise unspecified; M = male; PNFA = progressive non-fluent aphasia; SMD = semantic dementia.

* Gender information not available for one patient.

We reviewed available clinical notes of expansion-positive patients (Table 2). For those expansion carriers where the subtype of FTD was specified (n = 10), the most common diagnosis was bvFTD (n = 7, 70.0%) (Table 2). Two patients were diagnosed with combined FTD and ALS (FTD-ALS). One Spanish patient was diagnosed with PNFA. In the Spanish patient collection, the expansion was present in 4/73 (5.5%) of all bvFTD patients, and 3/10 (30%) of those bvFTD patients with a positive family history. The majority of expansion-positive patients had a positive family history of early-onset dementia or ALS (14/17, 82.4%). Of the three family history-negative patients, one reported early death of a parent, one reported a mother with anecdotal multiple sclerosis but no further clinical information available on this diagnosis, and one lacked sufficient family history information.

**Table 2:** Disease characteristics of 17 *C9ORF72* mutation carriers.

Group	Patient	Sex	Age at onset (y)	Clinical diagnosis	FH notes	FH score	Psychosis*	ALS*
Australia	1	M	52	Probable FTD	Twin ALS d41	3	No	(Yes)
Australia	2	M	43	bvFTD	Mother dementia d52	2	Yes	No
Australia	3	M	<57	bvFTD	Mother AD d67, aunt AD d74, uncle FTLD-U	2	N/A	(Yes)
Australia	4	M	59	bvFTD	10 relatives FTD and/or ALS	2	(Yes)	(Yes)
Australia	5	F	52	FTD-ALS	Sister, mother, uncle, grandmother dementia	1	N/A	(Yes)
Australia	6	M	65	FTD-ALS	8 relatives dementia or ALS	2	No	Yes
Australia	7	M	41	AD with R sided neglect	No FH (mother d<40)	4	N/A	No
Australia	8	M	55	FTD	Mother dementia d65	3	No	No
Australia	9	F	62	FTD	Brother ALS, mother dementia+scz	2	(Yes)	(Yes)
Australia	10	F	<64	FTD	Mother dementia d70	3	No	No
Australia	11	M	58	FTD	Mother, grandfather dementia d70; 2 relatives ALS	2	No	(Yes)
Australia	12	F	41	FTD	Brother dementia, mother ALS d 42	2	(Yes)	(Yes)
Spain	13	F	56	bvFTD	Brother DLB	3	N/A	No
Spain	14	M	64	bvFTD	-	4	No	No
Spain	15	F	63	bvFTD	Mother dementia	3	No	No
Spain	16	F	70	PNFA	(Mother multiple sclerosis)	4	Yes	No
Spain	17	M	51	bvFTD	Brother, father dementia	2	Yes	No

Data presented only for those *C9ORF72*-positive cases not previously reported. Details of six additional *C9ORF72*-positive cases have been reported previously [8].

AD = Alzheimer's disease, bvFTD = behavioural variant FTD, d41 = died at age 41, DLB = dementia with Lewy bodies, F = female, FH = family history, FTD-ALS = FTD with amyotrophic lateral sclerosis, M = male, PBP = pseudobulbar palsy, scz = schizophrenia; SMD = semantic dementia, N/A = data not available.

* Data for family members is in parentheses.

We examined the frequency of psychosis, ALS and parkinsonism, phenotypes commonly associated with FTD, in *C9ORF72* expansion carriers (Table 2). Psychosis was reported either in the proband or in an affected family member in 6/13 (46.2%) expansion-positive patients (4/9 Australian cases and 2/4 Spanish cases). ALS was reported either in the proband or in an affected family member in 8/12 (66.7%) Australian expansion-positive cases. Only five patients in the Spanish series had a personal or family history of ALS and none harboured the *C9ORF72* mutation. No *C9ORF72* expansion-positive FTD patients reviewed here reported a personal or family history of parkinsonism, although 1/6 *C9ORF72* carriers from this group that were reported previously [8] exhibited parkinsonian features.

### Segregation of repeat expansion

DNA was available from affected family members for four expansion-positive probands (cases 4 & 12 reported here and cases 97 & 105 reported previously [8]). For three families, segregation analysis showed that the expansion was present in all available affected members and absent in all available unaffected members. One of these families was previously reported to contain a mutation in the *SIGMAR1* gene (c.672*51G>T, family Aus-14) [14], [15]. We subsequently screened the other Australian *SIGMAR1* case (from family Aus-47, with *SIGMAR1* substitution c.672*26C>T) [15] and did not detect a *C9ORF72* expansion in this patient. For the fourth family, the mutation was present in the proband (diagnosed with bvFTD, onset age 56) but not his mother, who had developed dementia at age 68. When seen at age 78, the mother had clinical features much more suggestive of AD than FTD. In particular, in contrast to her son, she was not disinhibited or inappropriate, and had marked impairment in memory, language and orientation. The proband's father was said to have shown unusual behaviours and to have had several siblings with ALS and a parent with Parkinson's disease; these could not be confirmed at the time but in retrospect they were probably important.

We examined *C9ORF72* allele length in expansion-negative FTD patients (73 Australian, 94 Spanish) and healthy controls (275 Australian [8], 186 Spanish) to determine whether non-expansion alleles were associated with FTD phenotypes. We first examined whether demographic variables were correlated with the estimated expansion size. Using linear regression, gender was not significantly correlated with either mean or maximum repeat length in the Australian or Spanish cohort. Age was similarly not correlated with either mean or maximum repeat length in both cohorts. Mean and longest allele length were not associated with presence of disease in the Australian series (mean number of repeats ± SEM: cases 4.5±0.3, controls 4.4±0.1; longest repeat number: cases 6.0±0.4, controls 6.0±0.2; both *p*>0.05). However mean repeat length was significantly shorter in Spanish cases relative to controls (cases 4.1±0.2, controls 4.8±0.2, df = 224.5, *p* = 0.005). Maximum allele length was also significantly shorter in Spanish cases relative to controls (cases 5.5±0.3, controls 6.7±0.3, df = 221.0, *p* = 0.007). Mean allele length was not a significant predictor of age of onset in a linear regression analysis in either FTD group (Australian: β = 0.093, *t* = 0.780, *p* = 0.438; Spanish: β = −0.083, *t* = −0.785, *p* = 0.435). We repeated these analyses using longest allele length as a predictor, and again no significant associations were found.

### Expansion allele size estimation

**Figure 1:**
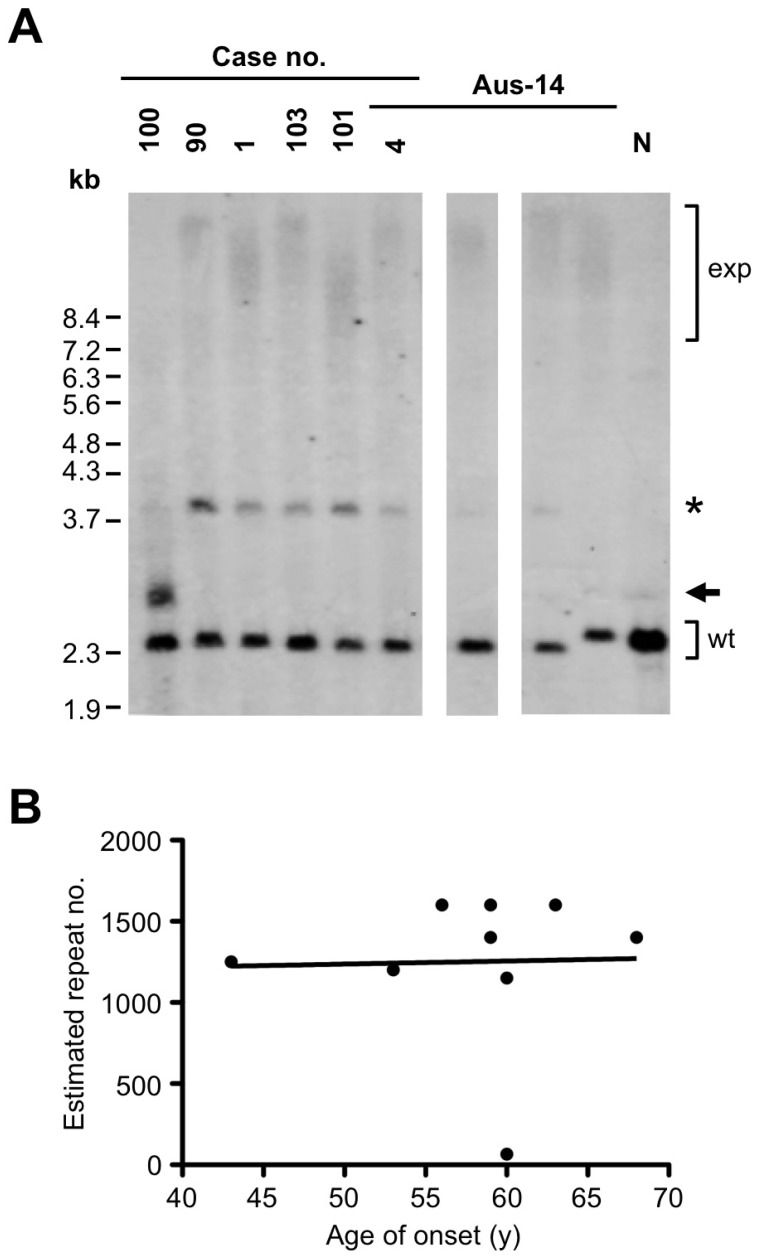
Analysis of *C9ORF72* expansion allele size. A, Southern blot analysis of five unrelated probands and four Aus-14 family members with the *C9ORF72* repeat expansion, plus an expansion-negative control (N). Wild-type (wt) and expansion (exp) alleles are indicated; the smaller expansion allele in case 100 is arrowed. A band running at ∼3.8 kb was also observed (asterisk); this probably arises from unspecific probe binding. B, Scatter plot of age of onset of disease versus estimated mean number of *C9ORF72* hexanucleotide repeats in expansion allele carriers.

We estimated the number of repeats in expansion alleles from nine Australian patients with sufficient DNA to perform Southern blotting: cases 1 & 4 reported here, plus three affected relatives of case 4 (IDs III-7, IV-1 & III-2 in family Aus-14 [14], [15]) and cases 90, 100, 101 & 103 from the neuropathologically ascertained cohort reported previously [8]. Eight patients harboured expansion alleles in the 1000–1600 repeats range (Figure 1A, Figure S1), within the range of expansion allele sizes reported previously [4]. In contrast, case 100 showed an estimated allele size ranging from 50–80 repeats (mean allele size ∼65 repeats). This man gave up driving at 53 because he was confused about which pedals to use. He stopped work at 58 and became socially withdrawn and egocentric, also developing delusions that the television was attached to his head or that fluid was running out of his legs. He developed incontinence and was admitted to residential care at age 66 years. He was treated with pericyazine and developed parkinsonian features, becoming increasingly impaired until his death at age 76. There was no report of symptoms consistent with ALS. Analysis of his brain post mortem led to a neuropathological diagnosis of frontotemporal lobar degeneration with type B TDP-43 pathology [8]. There was no family history of diagnosed dementia, although his mother was said to be vague when she died in a nursing home in her eighties. A half-sister had been treated with several courses of electro-convulsive therapy for a nervous breakdown. Genotyping of case 100 revealed that he was homozygous for allele A of rs3849942, the allele used as a surrogate marker for the chromosome 9p21 risk haplotype previously associated with FTD and ALS [11].

We examined demographic variables with respect to estimated expansion size. Mean estimated expansion size did not significantly differ between males and females (mean allele sizes ± SEM 1213±297 versus 1300±61, *p*>0.05). Linear regression analysis revealed no significant association between repeat number and age of onset (adjusted R^2^ = −0.142, β = 0.027, *t* = 0.073, *p*>0.05) (Figure 1B). This analysis was repeated with the case with the low repeat number excluded, but this did not affect the results.

## Discussion

We have screened two clinically ascertained patient collections for the hexanucleotide repeat expansion in *C9ORF72* that has recently been implicated in FTD and ALS [4], [5]. We identified expansions in 21 FTD probands, and determined the frequency of *C9ORF72* expansions to be 21–41% in familial FTD cases. Psychosis was a frequent observation in *C9ORF72* mutation carriers (46%), and, in the Australian series, the repeat expansion was common in FTD families with ALS features. We did not find any evidence for an increased prevalence of parkinsonism in *C9ORF72* mutation carriers, in agreement with some [16], [17], but not all previous reports [9], [10]. *C9ORF72* expansion patients were mostly of the bvFTD subtype: one carrier was identified with PNFA, which has been observed previously [5], [7], [9], [18].

The frequency of *C9ORF72* expansion in the Spanish familial FTD patient group is in line with recent estimates in European ancestry cohorts (2–19%) [6]. The relatively high frequency of *C9ORF72* mutations in the Australian patients is likely due to ascertainment bias, as this series was recruited by clinicians with an interest in early-onset dementia submitting blood for DNA analysis, where a clinical suspicion might be high for genetic mutation. In contrast, the Spanish subjects were recruited independently of positive FTD/ALS family history.

The exact size for the expanded allele in the *C9ORF72* is difficult to estimate by any other means than Southern blotting given our existing technology. As with other large expanded repeats, such as those for involved in myotonic dystrophies [19], Southern blotting typically reveal a smear corresponding to somatic instability within the cell population containing a range in size of the expanded allele (Figure 1, Figure S1). The presence of faint 3.9 kb band may have arisen as a result of the probe binding to homologous sequences, or subtle differences in DNA quality. The band was observed in all cases, with the exception of one affected member from the Aus-14 FTD-ALS family

Of interest is the finding that the Aus-14 FTD-ALS family that carries a mutation in *SIGMAR1* [15] also contains the *C9ORF72* repeat expansion (Case 4 in Table 2). The one family member presenting as a putative phenocopy, i.e. lacking the *SIGMAR1* variant, carries the *C9ORF72* repeat expansion. Thus, the most parsimonious explanation is that the *C9ORF72* expansion, and not the *SIGMAR1* mutation, is causative of disease in this family. However, the unrelated Australian *SIGMAR1* case reported in Luty et al [15] does not carry the *C9ORF72* repeat expansion. The identification by genome-wide homozygosity mapping of a *SIGMAR1* missense mutation in a family with autosomal recessive ALS [20] indicates that *SIGMAR1* may still play a role in the pathogenesis of ALS and FTD. In addition, recent studies reporting ALS patients with mutations in established causative genes (e.g., *SOD1*, *TARDBP*) in addition to *C9ORF72* [21]–[24] suggest that more than one pathogenic mutation may be required to cause disease in some ALS cases.

The *C9ORF72* repeat expansion largely segregated with disease in pedigrees where affected family members were available for testing, with one exception. We identified one family where the proband's late-onset affected mother (onset at age 68) was negative for the expansion. As we had access only to lymphocyte DNA from the mother we cannot rule out somatic mosaicism as a possible explanation for the negative finding, but given the paternal family history it seems more likely that she was a phenocopy. It should be noted the affected mother was homozygous for the ‘G’ allele of rs3849942 (data not shown), which is not associated with the common expansion haplotype [4], [5]. This lends further support that the mutant repeat expansion may not have been transmitted through her. Although the family history on the father's side was unconfirmed, in retrospect it was relevant. The identification of this probable phenocopy highlights the need to maintain a high index of suspicion when considering family histories, as the most obvious conclusion may be misleading.

We did not detect an association between *C9ORF72* allele length on FTD diagnosis or age of onset in the Australian patient group, implying that there is little pathogenic effect of a *C9ORF72* allele with fewer than 30 repeats. This is consistent with previous studies on FTD, ALS and FTD-ALS cohorts [8], [13], [25]. In our Spanish series, we detected a statistically significant increase in both mean and maximum allele length in controls relative to cases. However, it is premature to speculate on its significance until the biological mechanism behind the pathogenicity of *C9ORF72* allele length has been elucidated, and our findings replicated in independent cohorts of similar ethnic ancestry and disease status.

We did not detect a significant effect of expansion allele size on age of onset. However, the difficulty of accurate allele sizing with Southern blot analysis, most probably due to somatic mosaicism, and the small sample numbers available to us in this study mean that it is premature to draw conclusions from these findings. This analysis, however, did identify one patient with a *C9ORF72* allele comprising ∼65 repeats. To our knowledge, this is the smallest *C9ORF72* ‘expansion’ allele thus far reported in an FTD patient. This patient did not exhibit a late onset of disease, as might have been expected for someone harbouring a relatively small expansion. Studies have determined that most if not all *C9ORF72* expansion carriers share the same haplotype, implying that they are descended from a common ancestor [6], [11]–[13]. Recent discussion has centred on whether the *C9ORF72* expansion occurred only once on this haplotype and that this expansion has been inherited by all *C9ORF72*-positive patients, or if the haplotype represents an unstable *C9ORF72* allele that is prone to expansion, and has expanded to pathogenic lengths multiple times independently [12]. The detection of a *C9ORF72* allele with ∼65 repeats in an individual with the presumptive ‘risk’ haplotype is consistent with the ‘multiple expansion’ hypothesis.

In the absence of affected relatives to check for segregation with disease, it is not possible to determine conclusively whether the expansion to ∼65 repeats is causative of disease in this patient or merely a coincidental finding. However, in support of the former possibility, we note that this patient was given a neuropathological diagnosis of FTLD-TDP type B, the same pathology observed for the majority of *C9ORF72* expansion-positive cases [7]–[9], [26]. If truly pathogenic, this allele size may represent close to the minimum number of repeats necessary to cause disease. The minimum *C9ORF72* repeat number that invariably leads to FTD remains to be determined, as we and others have found a small number of apparently healthy controls with >30 repeats [8], [9], [23], and another study has reported reduced penetrance in some *C9ORF72* expansion-positive families [27]. Future large-scale Southern blot analyses of expansion-positive patients are required to establish exactly where the threshold for disease lies, as this information will have implications for genetic counselling for the substantial proportion of FTD patients who have been determined to be *C9ORF72* expansion positive by repeat-primed PCR.

## Materials and Methods

### Ethics statement

The procedures in this study were approved by the University of New South Wales and South Eastern Sydney Local Health District (Northern Region) Human Research Ethics Committees and the University of Navarra School of Medicine Ethics Committee. Written informed consent was collected from each participant or his/her guardian for brain donation and/or DNA collection.

### Subjects

We screened two patient groups: 91 patients from Australia and 99 from Spain (Table 1). All patients with *MAPT* or *GRN* mutations were previously excluded by standard DNA sequencing of the entire coding gene region.

The Australian patient group consists of patients recruited or referred for DNA studies of non-Alzheimer dementia since 1994, via specialist dementia clinics, neurologists, psychiatrists, geriatricians or genetic clinics. Patients were included if they had clinical features of FTD, dementia with frontal features, dementia otherwise atypical for AD, frontal neurodegenerative syndromes such as CBS or PSPS, and/or a family history of dementia or ALS. Of these 91 cases, 10 subsequently came to autopsy and formed part of the neuropathologically ascertained cohorts whose *C9ORF72* screening was reported previously [8]. Six of these 10 cases were positive for the *C9ORF72* expansion. The Spanish patient group was recruited at the Departments of Neurology of the following centres in Spain: Clínica Universidad de Navarra (Pamplona), Hospital do Salnés (Pontevedra) and Hospital de Bellvitge (Barcelona). It included independent FTD subjects diagnosed with sporadic or familial FTD.

Familial FTD was considered when the proband had a first-degree relative clinically diagnosed with dementia. Presence or absence of psychosis was evaluated by report from the referring clinician, based on neurologist and/or next-of-kin observations. Family history was scored according to a modified version of the Goldman scale [28], as detailed in Table S1.

We also screened 186 healthy Spanish controls with an average age of 64.2 years (range 19–87 years) at collection [29]. Australian patient allele sizes were compared with those from a cohort of 275 healthy Australian controls with an average age of 78.5 years (range 70–89 years), which was screened in a previous study [8].

### Mutation screening

Genomic DNA was screened for the hexanucleotide repeat expansion in *C9ORF72* by repeat-primed PCR, and non-expansion *C9ORF72* allele sizes were determined as described previously [8]. Southern blotting was performed on those patients where peripheral blood lymphocyte genomic DNA was available in sufficient quantities. DNA (10 µg) was digested with *Hind*III and *Xba*I and separated on a 0.9% agarose gel. Fragments were transferred by alkali blotting onto Amersham Hybond NTM-XL (GE Healthcare) and hybridized to a ^32^P-labelled probe at 70°C overnight. The filters were washed with Church's wash buffer (0.5 M sodium phosphate, 1% sodium dodecyl sulphate). Autoradiography was for 4–6 days at −80°C. *BstE*II digested lambda DNA was used as a size marker. Mean repeat number was used in statistical analyses.

### Statistical analysis

Statistical analyses were performed in IBM SPSS version 20. Non-expansion allele length was compared between cases and controls and expansion allele length was compared between males and females by means of Student's *t* test. We tested for homogeneity of variance by using the Levene statistic. When the Levene statistic was significant (*p*<0.05), the corresponding contrast estimate based on unequal variances was used. The effect of expansion and non-expansion allele length on age of onset was assessed by linear regression analyses. P-values<0.05 were considered to be statistically significant.

## Supporting Information

Figure S1 **Southern blot analysis of five unrelated probands and three Aus-14 family members with the** ***C9ORF72*** **repeat expansion, plus an expansion-positive control (+).** Wild-type (wt) and expansion (exp) alleles are indicated. The smaller expansion 2.9 kb allele in case 100 is arrowed. A band running at ∼3.8 kb was also observed (asterisk), probably arising from unspecific probe binding.
(TIF)

Table S1 **Modified Goldman scale for scoring family history.**
(DOCX)
