# Supplementary material for: C9ORF72 Repeat Expansion in Australian and Spanish Frontotemporal Dementia Patients
Source: PLoS One. 2013 Feb 20;8(2):e56899. doi: 10.1371/journal.pone.0056899 (PMC3577667; doi:10.1371/journal.pone.0056899)
Supplement: Table S1 — Modified Goldman scale for scoring family history. (DOCX) [file pone.0056899.s002.docx]

**Dobson-Stone et al**

***C9ORF72* Repeat Expansion in Australian and Spanish Frontotemporal Dementia Patients**

**Supplementary Material**

| Table S1 Modified Goldman scale for scoring family history | |
| --- | --- |
| Score | Criteria |
| 1 | ≥3 family members with FTD or associated disorders (CBS, PSPS, ALS) within two generations with one member being a first-degree relative of the other two |
| 2 | ≥2 family members with dementia or ALS but not meeting criteria for a score of 1 |
| 3 | 1 relative with FTD or early-onset (age <65 years) dementia or ALS, or 2 relatives in same lineage with late-onset dementia |
| 3.5 | 1 relative with late-onset (age >65 years) or unspecified dementia |
| 4 | No known family history |
| Abbreviations: ALS = amyotrophic lateral sclerosis; CBS = corticobasal syndrome; FTD = frontotemporal dementia; PSPS = progressive supranuclear palsy syndrome | |
